# Metformin and thyroid carcinoma incidence and prognosis: A systematic review and meta-analysis

**DOI:** 10.1371/journal.pone.0271038

**Published:** 2022-07-28

**Authors:** Zikun Wang, Juhua Luo, Yijia Zhang, Pengcheng Xun, Zhongxue Chen

**Affiliations:** 1 Department of Epidemiology and Biostatistics, School of Public Health, Indiana University Bloomington, Bloomington, Indiana, United States of America; 2 Department of Obstetrics and Gynecology and Department of Epidemiology, Columbia University Irving Medical Center, New York, New York, United States of America; Brigham and Women’s Hospital, Harvard Medical School, UNITED STATES

## Abstract

Metformin has been suggested to reduce thyroid cancer incidence and to improve thyroid cancer prognosis. We aimed to evaluate the associations between metformin and thyroid cancer incidence and prognosis (metastasis/recurrence/progression-free survival). Cochrane Library, PubMed, ClinicalTrials.gov, and U.S. National Library of Medicine Clinical Trials were searched through the end of December 2021. Data were collected from original observational studies or clinical trials on the incidence or prognosis of thyroid carcinoma outcomes in type 2 diabetes mellitus (T2DM) patients with and without metformin use. Risk of bias in non-randomized studies of interventions (ROBINS-I) tool and Grading of Recommendations, and Assessment, Development and Evaluations (GRADE) approach were used to evaluate the risk of bias and quality of the body of evidence, respectively. In general, 4 studies were related to the thyroid cancer incidence, including 1,705,123 participants metformin users and non-users and yielding a total of 3,238 thyroid cancer events; 3 studies reported the prognosis of thyroid carcinoma based on a total of 4,972 individuals with primary thyroid carcinoma and comorbid type 2 diabetes, and the number of thyroid cancer prognosis cases ranged from 3 to 79. The overall risk of bias of the included studies ranged from moderate to serious. In the random-effects model, the summary relative risk (SRR) for thyroid cancer incidence was 0.743 (95% CI: 0.453–1.220; I^2^ = 88.7%, low certainty) comparing metformin users to non-users; and SRR for the prognosis of thyroid cancer was 0.504 (95% CI: 0.178–1.430; I^2^ = 57.5%, low certainty). Non-statistically significant negative associations between metformin use and incidence and prognosis of thyroid cancer were found in the current analysis, although the quantity and quality of the evidence were limited. Futher investigation is needed to evaluate the clinical benefits of metformin on thyroid cancer prevention and treatments.

## Introduction

Thyroid carcinoma is one of the most common malignant endocrine tumors cancer in young women [1], and females are at higher risk of developing thyroid cancer compared to their male counterparts [2]. At a global level, the age-standardized incidence rate (ASIR) was increased by 20% from 1990 through 2013; [3] and the age-standardized death rate has also been increased by 31.7% since 1990 [4]. There will be 43,800 new cases of thyroid cancer in 2022 in the United States, and approximately 2,230 deaths from this disease in this year based on the report from the National Cancer Institute Surveillance, Epidemiology and End Result Program (SEER) [5]. Common risk factors for thyroid cancer include younger age (25–65 years old), female gender [2], non-Hispanics White [6], higher body mass index (BMI) [7, 8], being exposed to radiation during childhood [9], having a history of goiter (enlarged thyroid) [1], and having a family history of thyroid disease or thyroid cancer [5], such as familial medullary thyroid cancer (FMTC) [10].

Recently, a recognition that hyperinsulinemia is related to an increased risk of most cancers has been raised [11]. These patients with type 2 diabetes mellitus (T2DM) have long-term exposure to a high degree of glucose because of insulin resistance (IR), which can cause chronic inflammation [12]. Currently, associations between T2DM and the risks of several types of cancers, such as endometrial cancer, breast cancer, colorectal, lung, and prostate cancer, are well-known [13, 14]. The prevalence of thyroid disorders among people with diabetes (10.8%) is higher than that of the general population (6.6%) [15]. Moreover, many studies reported a positive association between T2DM and thyroid carcinoma [12, 16]. A meta-analysis conducted in 2014 concluded that the risk of thyroid cancer among patients with T2DM was increased by 34% (relative risk [RR] = 1.34;95% CI:1.11–1.63; P < 0.001), and T2DM was related to a 1.38 times increased risk of thyroid carcinoma in female (95% CI:1.13–1.67) in the sensitivity analysis [17]. Evidence was confirmed in another pooled analysis that thyroid cancer incidence was higher in females (RR = 1.11; 95% CI: 1.06–1.17; P-value < 0.001) who had T2DM in comparison with participants without T2DM [18].

Accordingly, these findings have led to a keen interest in antidiabetic drugs to prevent cancer. At present, several studies indicate that antidiabetic drugs, particularly metformin, could play a vital role in the development of carcinoma [19–22]. Metformin is a well-established first-line pharmacotherapy for the management of T2DM. This biguanide is an insulin sensitizer mainly produced in the liver and muscle. Some preliminary studies demonstrate that metformin can directly block CAF-driven NF-kB proinflammatory signals in order to compromise cancer progression [23]. Besides, HIF1 signaling can be blocked by increasing intracellular oxygen concentration, which leads to a subsequent reduction in tumor cell- driven VEGFA-mediated angiogenesis. On the other hand, metformin is considered to accelerate insulin sensitivity and decrease hepatic gluconeogenesis by affecting the insulin/IGF-I signaling pathway [24]. This is known to be the indirect carcinoma preventive mechanism of metformin [25]. Metformin can be activated at all sites with impaired insulin action [26]. It has various effects at different levels of the organs. For example, metformin raises insulin-mediated suppression of hepatic glucose production through reducing gluconeogenesis at the liver; it enhances insulin receptor phosphorylation, glucose transporter (GLUT)-4 translocation and leads to increased glucose uptake and glycogen synthesis in skeletal muscle; moreover, metformin promotes the re-esterification of free fatty acids and inhibits lipolysis in adipose tissue, which may indirectly promote insulin sensitivity through decreased lipotoxicity [26]. At present, many findings indicate that metformin can reduce the risk of a variety of cancers, such as lung cancer [20], bladder cancer [27], and endometrial cancer [28]. Furthermore, a meta-analysis summarizes that the summary relative risk (SRR) from cancers in patients who underwent metformin treatment declined approximately 31% (SRR = 0.69; 95% CI: 0.61–0.79) in both incidence and mortality, compared to patients without metformin treatment [25].

Although some evidence indicates that metformin may decrease the incidence and the prognosis of thyroid cancer, such as metastasis, recurrence, and progression-free survival, findings from other studies did not support the hypothesis [29–31]. Moreover, a thorough systematic and quantitative assessment of literature related to metformin and thyroid cancer is not available at present. Thus, given the current situation, we aimed to conduct a meta-analysis and to assess the strength of the evidence supporting the association of metformin use in relation to thyroid cancer incidence and prognosis of thyroid cancer in patients with T2DM.

## Materials and methods

### Information sources and search strategy

The search was restricted observational studies and clinical trials. The PRISMA checklist was applied to guide the analysis’s procedure when developing a systematic review protocol for the current analysis [32]. Of these studies, the thyroid cancer incidence among diabetic patients with metformin treatment was compared with those who were not treated with metformin. Moreover, for thyroid cancer prognosis, studies that had reported thyroid cancer with metastasis, thyroid cancer recurrence, and thyroid cancer progression-free survival among patients who had primary thyroid carcinoma and comorbid T2DM with metformin treatment were compared to patients who were non-metformin users were included. A systematic review was conducted in the following databases: the Cochrane Library, PubMed, ClinicalTrials.gov, and the U.S. National Library of Medicine Clinical Trials. Then, the following keywords were adopted: “Diabetes Mellitus” AND “Biguanides” AND “Diabetes Treatment” OR “Metformin” AND “Thyroid Cancer” OR “Thyroid Carcinoma” for journals. After the search terms and databases were agreed upon, a comprehensive search was performed for each database, and all records were collected. A preliminary review of the titles and abstracts was made to determine whether the article was relevant to our topic. In addition, bibliographies were reviewed for retrieving relevant studies. We also did a manual search for references cited in the selected articles. The authors’ names of all selected publications were eliminated first to reduce the potential bias in selecting articles, data extraction, and quality assessment.

### Inclusion and exclusion criteria

The following predefined inclusion criteria were incorporated in the meta-analysis: (1) in human; population independent from other studies to avoid giving double weight to estimates derived from the same study; (2) patients who were treated with metformin alone or in combination with other antidiabetic treatments; (3) the outcomes of interest were incidence and prognosis of thyroid carcinoma measured by the occurrence of thyroid carcinoma, the metastasis, the recurrence-free survival, and the progression-free survival (disease progression survival) of thyroid cancer, respectively; (4) studies have adequate information to assess the relative risk estimates (Odds ratios (ORs)/Hazard ratios (HRs)) with the 95% confidence intervals (CI); or studies were reported such information which could be derived based on the reported results or obtained by contacting the original authors; (5) original observational studies (case-control study or cohort study) or the clinical trials with any duration of follow-up; (6) published full-text report; in English-language; (7) if more than one study were published from the same cohort and objectives, the data were collected from the one with the most detailed information for both outcomes and exposure, or the one with the most comprehensive population; and (8) the search was limited to articles published from January 1^st^, 1970, to December 1^st^, 2021.

**Fig 1:**
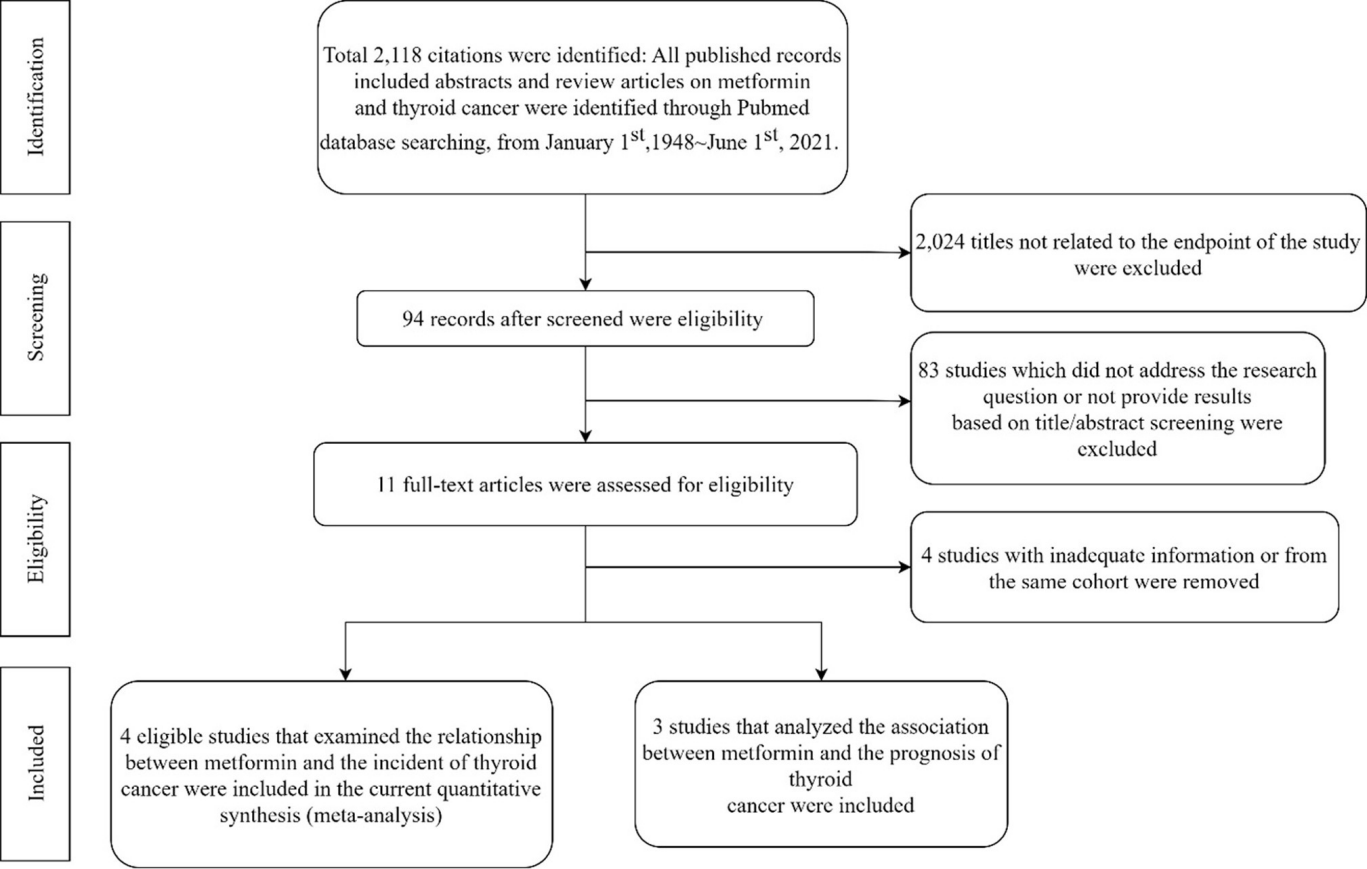
Flowchart of the study selection process.

The exclusion criteria were: (1) patients with type I diabetes only; (2) abstract, case report, review, meta-analysis, and letter to the editor; and (3) a lack of explicit methodology and results, duplicate publication. A PRISMA flow diagram for the study selection in the meta-analysis was summarized in Fig 1.

### Study selection and data extraction

Data were reviewed and extracted by two authors. (ZW, YZ) Full-text or report of each study was reviewed to decide its eligibility and then tabulated all the relevant data independently, the discrepancies of data extraction between two authors were resolved by discussion and consensus. The extracted data from each selected article was collected and summarized as (1) general information (authors’ name, publication year, and study location); (2) study design; (3) sample size (cases and controls or cohort size); (4) exposure ascertainment: (Ever used metformin by medical records/insurance records/self-reported questionnaires); (5) comparison group (Non-antidiabetic drug use/Never used metformin); (6) primary outcome (incidence or prognosis of thyroid cancer): a clearly stated diagnosis of thyroid cancer; or thyroid cancer with metastasis or thyroid cancer recurrence-free survival or thyroid cancer progression-free survival; (7) Adjusted variables included in analyses, such as age, gender, BMI, other antidiabetic drugs used, the severity/duration of diabetes, smoking, education level, comorbidity, previous thyroid diseases, and etc. The summary of the characteristics of the included studies is displayed in Table 1.

**Table 1:** Characteristics of studies included in the systematic review.

Study, and location	Study design	Total No. of Exposed/Unexposed ^a^	No. of new Thyroid cancer cases in the exposed/the unexposed group	Source of diagnosis	Metformin Dose/Duration	Multiple adjusted ORs/HRs (95% CI)	Adjustments	Risk of bias assessment ^c^
^b^ Tseng CH (2014), [33] China.	Retrospective cohort study	795,321/619,402	683/1,614	NHI records	Cumulative dose (mg)>126,3000, 264,000–1,263,000, <264,000/ Cumulative duration (months): <9.13, 9.13–37.00 >37.00	HR:0.432 (0.336~0.555)	Age, the severity/duration of diabetes, other antidiabetic drugs used, hypertension, gender, other cancer, chronic diseases status, medication.	Moderate
^b^ Luo JH et al (2016), [29] USA. ^d^	Prospective cohort study	6,411/26,594	9/35	Self-reports	-/-	HR:0.985 (0.458~2.120) ^e^	BMI, smoking, previous thyroid diseases, age, race, education, smoking, physical activity, alcohol, hormone treatments, previous thyroid disease.	Moderate
^b^ Cho YY et al (2018), [34] Korea.	Prospective cohort study	128,453/128,453	340/487	Public medical insurance records	868,169 (±563,221) mg/ 1,633(±915) days	HR:0.690 (0.600~0.790)	Age, income, living area, gender, other antidiabetic drugs used.	Moderate
Study, and location	Study design	No.of Case/control	No.of Exposed in cases/controls	Source of diagnosis	Metformin Dose/Duration	Multiple adjusted ORs/HRs (95% CI)	Adjustments	Risk of bias assessment
^b^ Becker C et al (2015), [30] UK.	Case-Control study	70/419	47/254	Medical READ codes	-/-	OR:1.420 (0.740~2.730)	BMI, smoking, the duration of diabetes, other antidiabetic drugs used, previous thyroid diseases.	Moderate
Study, and location	Study design	Total No. of Exposed/Unexposed a	No. of new Thyroid cancer cases in the exposed/the unexposed group	Source of diagnosis	Metformin Dose/Duration	Multiple adjusted ORs/HRs (95% CI)	Adjustments	Risk of bias assessment c
^f^ Klubo-Gwiezdzinska J et al (2013), [35] USA.	Retrospective Cohort study	34/21	6/9	Hosptial medical records	500–1,000 mg/day, 1,000–1,500 mg/day, 1,500–2,000 mg/day > 2,000 mg/day/4.4±3 years	HR:0.109 (0.0184~0.640)	Age, gender, other antidiabetic drugs used, BMI, tumor size, RAI activity, gross extrathyroidal extension, presence of lymph node metastases, presence of distant metastases.	Serious
^f^ Jang EK et al (2015), [36] Korea.	Retrospective cohort study	35/25	3/3	Records from Asian medical center	Mean dose: 979 mg/day /Mean duration of metformin: 7.4 ± 4.8 years	HR:0.714 (0.157~3.252) ^g^	—	Serious
^f^ Noh Y et al (2019), [31] Korea.	Prospective cohort study	2,449/2,408	56/79	Records from HIRA (Korean)	-/-	HR:0.790(0.560~ 1.120)	Age, diabetes history, BMI, gender, other antidiabetic drugs used, comorbidity.	Moderate

Abbreviation: *NHI*, *Taiwan’s national health insurance; HIRA*, *The Health Insurance Review and Assessment Service*.

^a^: Exposure ascertainment: (Ever used metformin by medical record/Self-reported metformin use); Comparison group (Non-antidiabetic drug use/Never used metformin);

^b^: Incidence study;

^c^: Risk Of Bias In Non-randomized Studies of Interventions (ROBIN-I) tool; [37]

^d^: Study was conducted among females;

^e^: Hazard ratio was provided by the original authors through a personal communication for a comparison between ever used metformin/never used metformin among females with diabetes;

^f^: Prognosis study (metastasis/recurrence/progression-free survival);

^g^: Crude (unadjusted) hazard ratio and 95%CI were calculated based on the numbers provided in the original study.

### Risk of bias and certainty of the evidence

Risk of bias assessment was conducted by paired authors (ZW, YZ) independently using the Risk Of Bias In Non-Randomized Studies of Interventions (ROBINS-I) tool [37]. Accordingly, seven different domains were included, such as bias due to confounders and selection of participants for the study, bias because of classification of interventions, deviations from intended interventions, bias of missing data, bias due to measurement of outcomes and selection of the reported result. The risk of bias on the study level was evaluated and graded as low (the study can be considered comparable to a well-performed randomized trial), moderate (the study is robust for a non-randomized study, but it cannot be compared to a well-conducted randomized study), serious (there are some important problems in the study), and critical risk of bias (little empirical evidence on the effects of intervention exists in the study because the study is too problematic) for each study; specifically, a study was graded as “low risk of bias” if each domain was rated as low; a study was defined as “moderate risk of bias” or “low-to-moderate risk of bias” if one or more domains were rated as “probably at risk”; a study was rated as “serious risk of bias” if one or more domains were judged to be “serious risk of bias”; and a study was rated as “critical risk of bias” if it was rated as ‘critical risk’ in at least one domain.

The Grading of Recommendations Assessment, Development and Evaluation (GRADE) approach [38] was applied to assess the certainty of evidence on the confidence in the estimates of effect for each outcome as very low, low, moderate, or high, based on considerations of risk of bias, inconsistency, indirectness, imprecision, and other considerations (ie, magnitude of effect, dose-response effect, and impact of residual confounding and bias). Accordingly, very low certainty indicates that the studies have very limited confidence in effect, whereas the high certainty means the evidence found in studies is very likely to be true effects. In the GRADE approach, the observational studies are first graded as low-quality evidence and they can be rated upgrade if the observational studies provide a large magnitude of effect, or dose-response trend.

### Synthesis methods and reporting bias assessment

Summary risk ratio (SRR), defined as the exponent of the inverse-variance weighted mean of the natural logarithm of the relative risk estimates (HRs/ORs) [39], with theirs corresponding 95% confidence intervals (CIs), was used as a pooled effect measure of the association of metformin treatment with the incidence/prognosis of thyroid cancer. If multiple HRs/ORs were displayed in studies, the estimates were extracted from the analyses’ final model. Extracted data were presented in Table 1 as well.

The heterogeneity across studies was evaluated using *I*^2^ index; [40] *I*^2^ ≥ 50% and P-value <0.05 were considered heterogeneous among studies; a fixed-effect model was used to estimate the pooled SRR when *I*^2^ < 50%; while the random-effects model was adopted if *I*^2^ ≥50%. Forest diagrams were plotted to present the pooling of individual risk ratios to estimate SRRs for all thyroid cancers. Additionally, Egger’s linear regression [41] was conducted to test the bias formally, and a funnel plot was used to present the publication bias visually, and an asymmetric funnel plot was regarded as an indication of possible publication bias [42].

Statistical analyses were conducted using the statistical software R version 3.5.2. (http://www.Rproject.org/) (R Core Team, Vienna, Austria) using the R package “metafor.” [43] P-value <0.05 was considered statistically significant. Additionally, the *robvis* tool was applied to create risk-of-bias plots; [44] and the GRADEpro app was used to rate evidence and to generate the evidence profiles and summary of findings [45].

## Results and discussion

A total of 2,119 articles were identified from the electronic search, of which 2,025 were excluded because of irrelevant information after title/abstract screening. After full-text reading, 81 articles were excluded because of case report and review articles (n = 5), not addressing the research questions (n = 72), and not human studies (n = 4). Three studies from the same cohort [46–48], and one study with missing information on outcomes of interest [49], which did not succeed in contacting the authors to retrieve, were excluded, and nine studies remained. Two intervention studies [50, 51] from ClinicalTrails.gov were further excluded because no available results were presented. A manual search of the reference lists of these studies yielded no additional eligible studies. Finally, a total of seven observational studies were included in this meta-analysis [29–31, 33–36]. To have a better comparison, HR from Luo JH et al. (2016) was provided by the original authors through a personal communication for a comparison between metformin users and non-metformin users among females with T2DM; [29] and the crude (unadjusted) HR and 95%CI from Jang EK et al. (2015) were calculated based on the numbers provided in the original study [36].

### Characteristics of all studies

Among all seven included studies (one case-control study and six cohort studies), three were conducted in Korea, one in China, two in the United States, and one in the U.K. Six of the seven studies applied adjusted multivariable analyses; [29–31, 33–35] Age [29–31, 33–36], gender [30, 31, 33–36], and BMI [29–31, 35] were adjusted in most of the included studies. In addition, two incidence studies [30, 33] adjusted for the severity/duration of diabetes; other antidiabetic drugs used were adjusted for in three incidence studies [30, 33, 34] and two prognostic studies; [31, 35] and previous history of thyroid diseases were controlled for in two other incidence studies [29, 30].

### Metformin and thyroid cancer incidence

**Fig 2:**
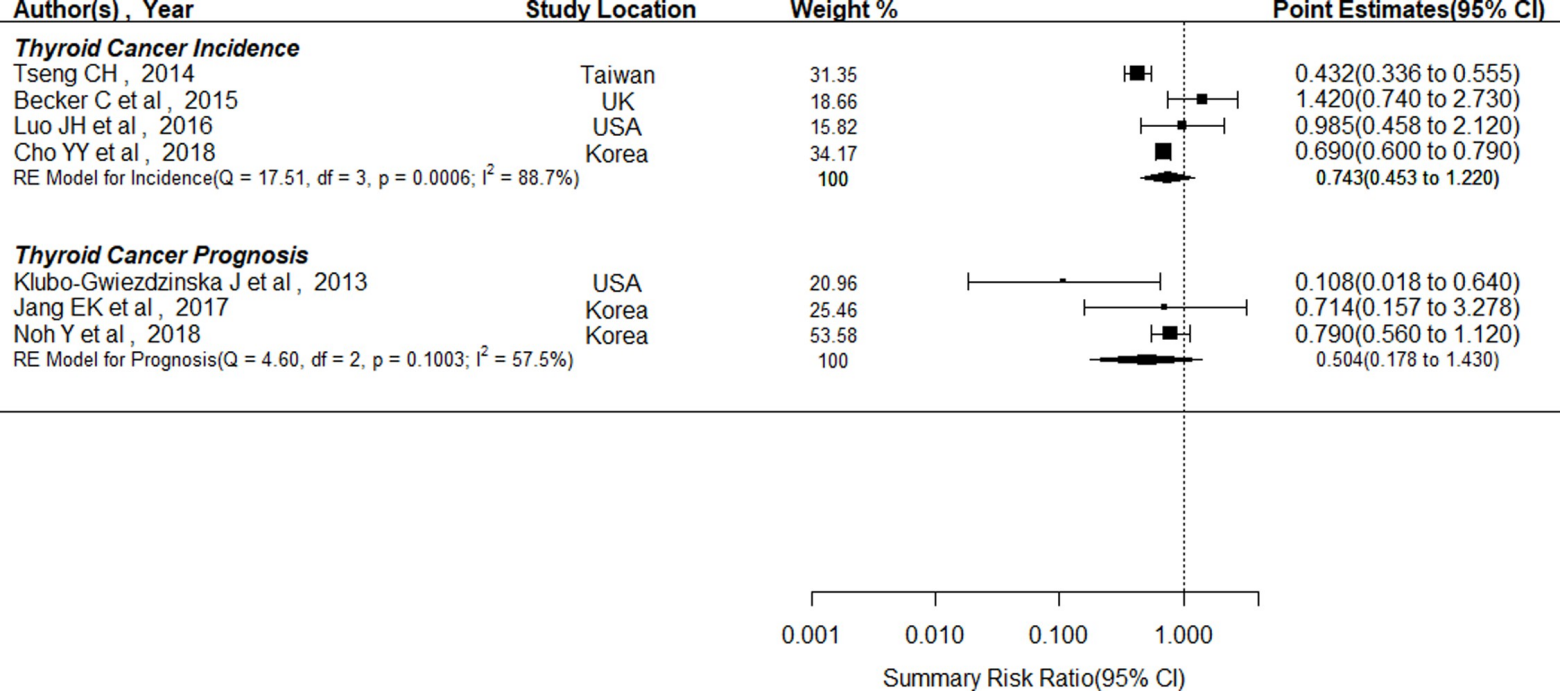
Forest plot of associations between metformin, incidence and prognosis of thyroid cancer^a,b^. ^a^: Black boxes denote point estimates for HRs and ORs, and horizontal lines represent 95% CIs. Black diamonds, Summary risk ratio (SRR) estimates. No association under the null hypothesis (risk estimate equal to 1.00) was represented by the vertical dotted line. ^b^:Incidence studies: [29, 30, 33, 34]; Prognosis studies: 1) Klubo-Gwiezddzinska J et al, [35] the progression-free survival of thyroid cancer; 2) Noh Y et al, [31] the metastasis of thyroid cancer; 3) Jang EK et al, [36] the recurrence of thyroid cancer.

Four studies examined the incidence of thyroid cancer [29, 30, 33, 34], totalling 1,705,123 participants (930,486 metformin users and 774,637 non-users) and yielding a total of 3,238 thyroid cancer events. The SRR of thyroid cancer showed that metformin was non-significantly associated with a decrease in the occurrence of thyroid cancer (SRR = 0.743; 95% CI: 0.453–1.220), and these results are presented in Fig 2. Additionally, the heterogeneity was assessed, and Higgins *I*^*2*^ reflected 88.7% heterogeneity (p-value = 0.0006). The funnel plot conducted by Egger’s test (S1 Fig) indicated no potential publication bias (p-value = 0.1528) for four incidence studies.

### Metformin and the prognosis of thyroid cancer

Three studies assessed the prognosis of thyroid carcinoma with a total of 4,972 patients who had thyroid cancer with metastasis [31], or thyroid cancer recurrence and comorbid T2DM [36], or patients who reported thyroid cancer progression-free survival and comorbid T2DM [35]. The number of prognostic cases (metastasis/recurrence/progression-free survival) ranged from 3 to 79. The SRR indicated that patients seemed to had a better prognosis of thyroid cancer among metformin users (SRR = 0.504; 95% CI: 0.178–1.430), compared to non-metformin users and non-antidiabetic drug users in the random-effects model. However, a relatively wide range of 95%CI suggested this relationship was not statistically significant; and between-study heterogeneity was not statistically significant (I^2^ = 57.5%, p-value = 0.1003), as well. All of these findings are presented in Fig 2. Accordingly, the results suggested that no potential publication bias existed for the prognostic studies based on Egger’s test (p-value = 0.3300). The results of the funnel plot were presented in S2 Fig.

### Risk of bias and certainty of the evidence

**Fig 3:**
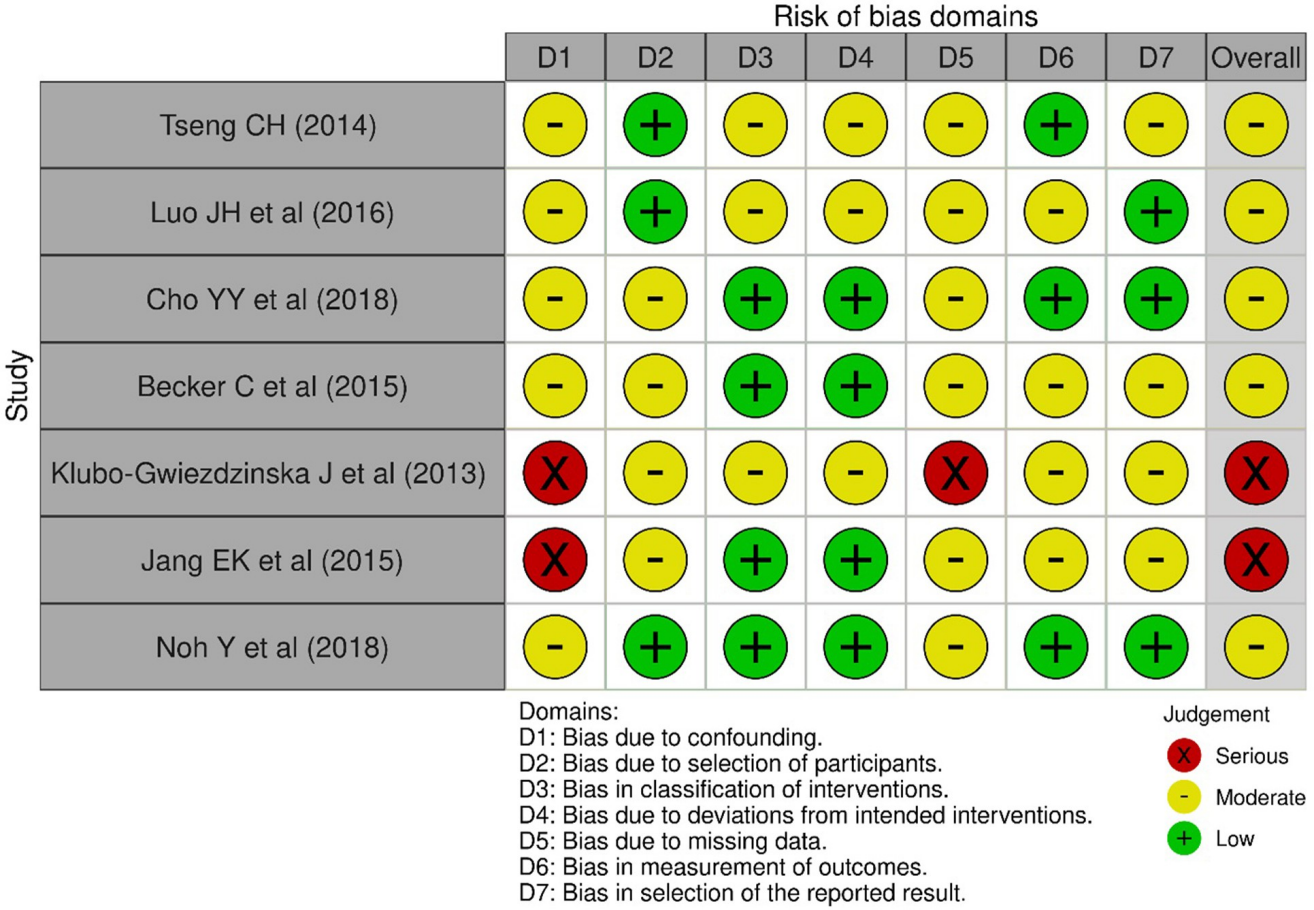
The risk of bias assessment for each study.

Despite the risk of bias for both incidence and prognosis of thyroid cancer studies ranged from moderate to serious according to the ROBINS-I Assessment approach (Table 1), the quality of evidence overall for most studies was at moderate risk of bias for seven domains, and the main sources of bias were associated with missing data and confounding controls (such as lifestyle-related factors and comorbidity). Fig 3 summarizes the evaluations for each domain across full reported studies. The overall certainty of the evidence for thyroid cancer incidence and prognosis studies were low certainties based on the GRADE approach since all included studies were observational studies. GRADE evidence profile for the summary of findings is presented in Table 2.

**Table 2:** GRADE evidence profile, ^a^ and summary of findings on metformin and thyroid cancer incidence and prognosis.

Certainty assessment							Summary of findings			
No. of studies/Study design	Risk of bias	Inconsistency	Indirectness	Imprecision	Other considerations ^b^	Overall certainty of the evidence	No. of participants		Summary Relative effect (95% CI)	Plain-Language Summary
							With Metformin	Without Metformin		
Incidence of Thyroid Cancer										
4/observational studies	not serious	not serious ^c^	not serious	not serious	none	⨁⨁◯◯Low	1,079/930,255 (0.1%)	2,381/774,868 (0.3%)	0.743 (0.453–1.220)	Metformin intake may result in a decrease in thyroid cancer incidence, but there was insufficient evidence to support the benefit of metformin on the risk of thyroid cancer.
Prognosis of Thyroid Cancer (metastasis/recurrence/progression-free survival (disease progression survival))										
3/observational studies	not serious	not serious	not serious	not serious	none	⨁⨁◯◯Low	65/2,518 (2.6%)	91/2,454 (3.7%)	0.504 (0.178–1.430)	Metformin intake may result in a large reduction in thyroid cancer prognosis, but there was insufficient evidence to support the benefit of metformin on the prognosis of thyroid cancer.

^a^: Table based on the Grading of Recommendations Assessment, Development and Evaluation (GRADE) approach [45], and all studies were non-randomized and evaluated using non-randomized studies of interventions (ROBINS-I) tool;

^b^: Other considerations included magnitude of effect, dose-response effect, and impact of residual confounding and bias

^c^: case-control and other study designs together.

In general, the current meta-analysis of observational studies assessed the associations of metformin use with the development and prognosis of thyroid cancer; specifically, non-statistically significant negative associations between metformin use and incidence and prognosis of thyroid cancer were found; and the certainty of the evidence was low due to imprecise estimates, heterogeneity across the studies, and risk of bias in the assessment of outcomes.

Previous research suggested that an association has been observed between increased IR and the risks of several common carcinomas, including differentiated thyroid carcinoma (DTC) [52, 53]. Clemmons, D. R. [54] explained that insulin plays a vital role in cell proliferation and apoptosis because it affirms the receptors with IGF-I, and it could share structural homology with IGF-I as well. Besides, the elevation of insulin and IGF-1 levels in T2DM will stimulate insulin receptors on neoplastic cells and promote cancer growth and division [55]. Additionally, Rezzonico et al. showed that IR was presented in 50% of DTC, but only in 10% of the control group (P-value < 0.001) [56]. Moreover, some evidence supports that metformin could regulate insulin sensitivity improvement and could potentially result in a decline of insulin resistance [57]. Furthermore, two popular hypotheses tried to explain the potential anticancer properties of metformin in the preclinical model; [58] these mechanisms include the improvement of insulin sensitivity, as well as the activation of adenosine 5’-monophosphate-activated protein kinase (AMPK) pathway [59]. However, the mechanisms of these potential antineoplastics of metformin for thyroid cancer are not yet fully clarified.

Although increasing evidence indicates a potential antitumor effect of metformin in thyroid cancer cell lines in vitro [60, 61], results from the pooled analyses of observational studies indicated that non-statistically significant decreases in incidence and prognosis of thyroid cancer were observed among metformin users compared to non-users. However, the current findings should be interpreted with caution in case of the inherent bias of the observational study. Overall, the risk of bias for most studies was graded to be moderate, and the certainty of the evidence was low for the incidence, metastasis, recurrence, and progression-free survival of outcomes due to the moderate risks of bias and limitations in the study design. Heterogeneity in the current analysis existed as well as in types of study setting (population-based vs. hospital-based), the sample size of studies, particularly the prognostic studies, types of participants (Asian vs. Western), interventions (different effects of other antidiabetic medicines), and outcome measurements [62]. Besides, it is plausible that we only have a limited number of incidence and prognostic studies, which could not find a significant effect. Moreover, all of our analyses were based on observational studies, and immortal time bias cannot be avoided [63]. In pharmacoepidemiology, immortal time bias refers to “a period of cohort follow-up time during which death (or an outcome that determines the end of follow-up) cannot occur” [63]. Currently, immortal time bias could be generated when studies estimated “any metformin use.” Therefore, person-time survived until metformin initiation is then erroneously attributed to metformin. Additionally, the Korean study also indicated a beneficial effect among thyroid cancer patients who used metformin in later periods rather than in early periods [34]. However, we did not evaluate the dose-response effect due to the limited number of studies.

Many preclinical studies [60, 64, 65] mentioned that metformin was related to cell growth inhibitory because of cell proliferation inhibition instead of induction of cell apoptosis or necrosis. This evidence indicated that patients who had monotherapy with metformin might not lead to complete remission of the carcinoma. However, we were not able to adjust all other treatments for thyroid cancer. Although the reduced cancer incidence and better prognosis are expected by comparing metformin and other antidiabetic or no antidiabetic treatment according to previous evidence, more studies on whether metformin could reduce thyroid cancer risk or whether metformin users could have better prognoses among patients with thyroid cancer should be further explored.

The present meta-analysis has several strengths. To the best of our knowledge, no other meta-analysis has been conducted on the association of metformin use with the incidence of thyroid cancer, and the association of metformin with the prognosis of thyroid carcinoma. In addition, most included studies applied to adjusted multivariable analyses, and several important confounders, including age, BMI, the severity/duration of diabetes, other antidiabetic drugs used, and comorbidity, were adjusted for when evaluating the associations between metformin, incidence and prognosis of thyroid cancer in specific populations. Moreover, this study used meta-analysis, a powerful quantitative synthesis skill of data allowing aggregation of studies with similar methodologies and endpoints. Furthermore, we assessed the risk of bias in studies meeting predefined inclusion and exclusion criteria by applying the ROBINS-I tool, and most of the studies were rated as having a moderate risk of bias.

Nonetheless, some limitations should be considered. Half of the included studies were based on Real-world data (e.g., insurance records or self-administered questionnaires) that was not explicitly designed to assess the association of metformin therapy on thyroid carcinoma. Therefore, possible selection bias from insurance records and recall bias of self-reports cannot be ruled out in our study, and the estimated effects might be biased away from or towards the null [66]. Besides, these included literature all published in English, and we only focused on the published studies and excused the “grey literature” (such as theses and conference abstracts). Therefore, potential publication bias due to language limitation and other factors could still be an issue in the current meta-analysis. Moreover, meta-analysis methods have limited performance with a small number of studies. In addition, great heterogeneity exists for population demographics, and follow-up duration; Besides, the studies that were included in the evaluation of prognosis had relatively smaller sample sizes compared to those that were evaluated in the incidence studies, which might lead to bias. Although our analyses were obtained from appropriate meta-analytic techniques with random-effect models, it was hard for us to account for these differences. Furthermore, given the limitation of the data, lower certainty of effects were reported for included studies regardless of incidence and prognosis of thyroid cancer.

## Conclusions

Overall, the quantity and quality of the evidence were limited in the current analysis, and non-statistically significant inverse associations between metformin use, the incidence and prognosis of thyroid cancer were observed. Further rigorous research, especially well-designed prospective studies with large sample sizes, is needed to disentangle the roles of metformin in thyroid cancer prevention and treatments.

## Supporting information

S1 Fig Funnel plot testing for the publication bias on the association between metformin and thyroid cancer incidence. (TIF)

S2 Fig Funnel plot testing for the publication bias on the association between metformin and thyroid cancer prognosis. (TIF)

S1 Table Detailed of the risk of bias in non-randomized studies of interventions (ROBINS-I) for each included cohort study. (DOCX)

S2 Table Detailed of the risk of bias in non-randomized studies of interventions (ROBINS-I) for each included study. (DOCX)

S1 File Detailed of the risk of bias in non-randomized studies of interventions (ROBINS-I) for each included study scores. (DOCX)

S2 File PRISMA checklist. (DOCX)
