# Supplementary figures and images for: Metformin and thyroid carcinoma incidence and prognosis: A systematic review and meta-analysis
Source: PLoS One. 2022 Jul 28;17(7):e0271038. doi: 10.1371/journal.pone.0271038 (PMC9333305; doi:10.1371/journal.pone.0271038)

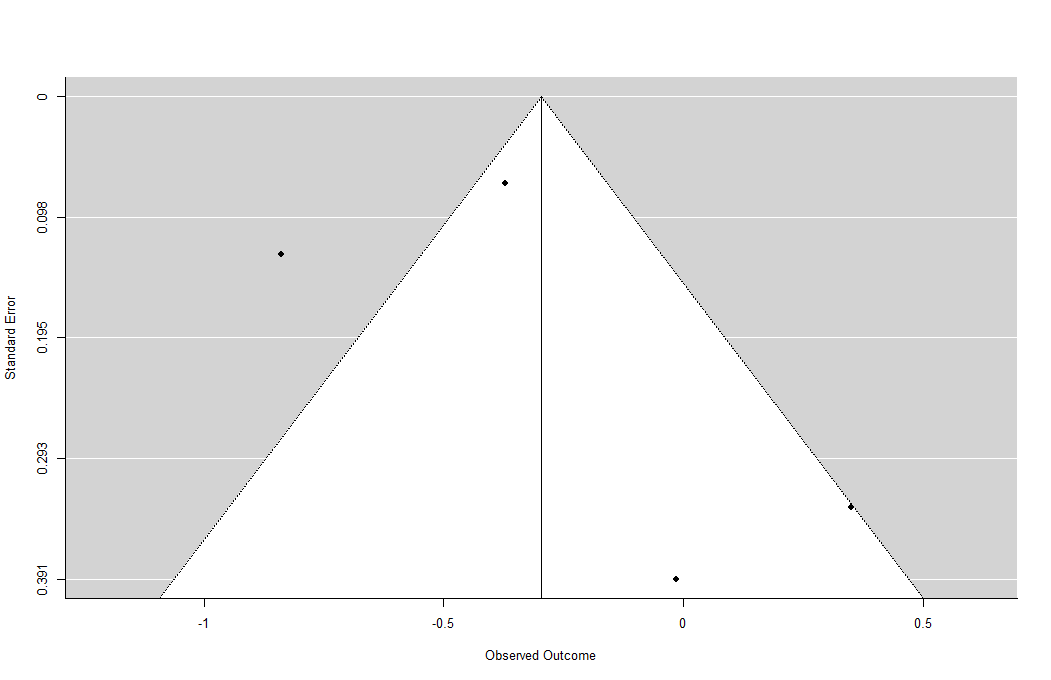

Supplement: S1 Fig — (TIF) [file pone.0271038.s001.tif]

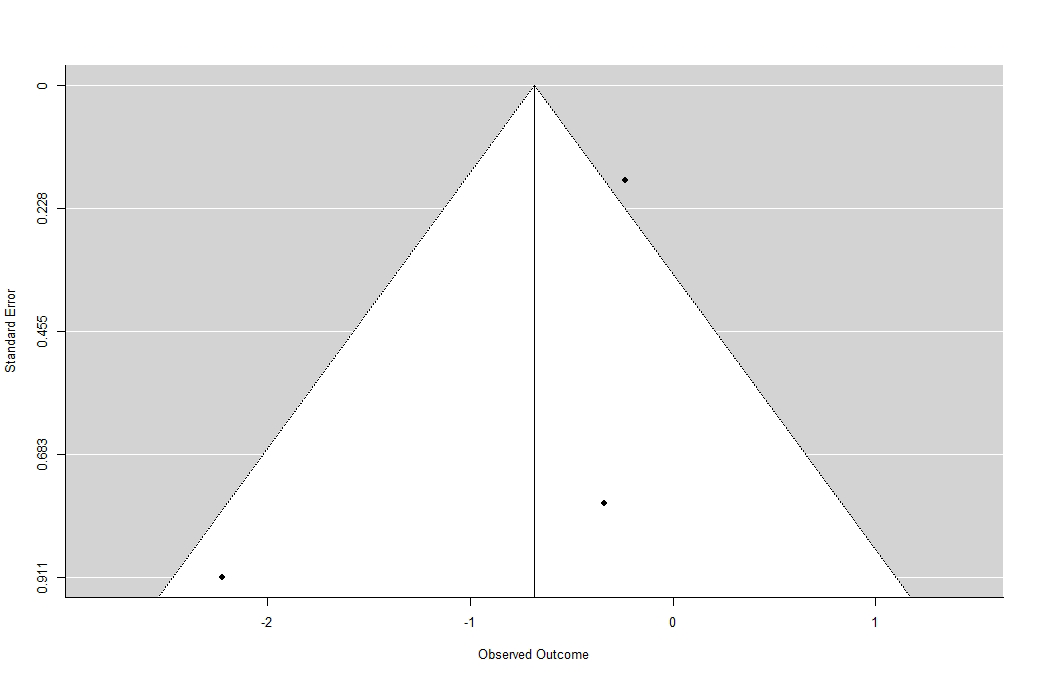

Supplement: S2 Fig — (TIF) [file pone.0271038.s002.tif]
