# Supplementary material for: Metformin and thyroid carcinoma incidence and prognosis: A systematic review and meta-analysis
Source: PLoS One. 2022 Jul 28;17(7):e0271038. doi: 10.1371/journal.pone.0271038 (PMC9333305; doi:10.1371/journal.pone.0271038)
Supplement: S1 Table — (DOCX) [file pone.0271038.s003.docx]

***S1 Table: Detailed of the Risk of Bias ^a^ in Non-randomized Studies of Interventions (ROBINS-I) for Each Included Study ^b^***

| **Study** | **Bias due to confounding** | **Bias in selection of participants into the study** | **Bias in classification of interventions** | **Bias due to deviations from intended interventions** | **Bias due to missing data** | **Bias in measurement of outcomes** | **Bias in selection of the reported result** | **Overall** |
| --- | --- | --- | --- | --- | --- | --- | --- | --- |
| Tseng CH (2014) | Moderate | Low | Moderate | Moderate | Moderate | Low | Moderate | Moderate |
| Luo JH et al (2016) | Moderate | Low | Low | Moderate | Moderate | Low | Low | Moderate |
| Cho YY et al (2018) | Moderate | Moderate | Low | Low | Moderate | Low | Low | Moderate |
| Becker C et al (2015) | Moderate | Moderate | Low | Low | Moderate | Moderate | Moderate | Moderate |
| Klubo-Gwiezdzinska J et al (2013) | Serious | Moderate | Moderate | Moderate | Serious | Moderate | Moderate | Serious |
| Jang EK et al (2015) | Serious | Moderate | Low | Low | Moderate | Moderate | Moderate | Serious |
| Noh Y et al (2018) | Moderate | Low | Low | Low | Moderate | Low | Low | Moderate |

^a^_:_ According to Risk Of Bias In Non-randomized Studies of Interventions (ROBINS-I):[34] Low risk of bias means “the study is comparable to a well-performed randomized trial with regard to this domain)”; Moderate risk of bias means “the study is sound for a non-randomized study with regard to this domain but cannot be considered comparable to a well-performed randomized trial”; Serious risk of bias means “the study has some important problems in this domain”; Critical risk of bias means “the study is too problematic to provide any useful evidence on the effects of intervention in this domain”.

^b^_:_ The quality of included studies was assessed by Author 1.
